# Outbreaks of COVID-19 in a tuberculosis treatment sanatorium on the Thailand-Myanmar border: a retrospective cohort analysis

**DOI:** 10.12688/wellcomeopenres.19275.2

**Published:** 2023-11-01

**Authors:** Htet Ko Ko Aung, Lei Lei Swe, Makoto Saito, Sophie Lesseps, Naw Janurian, Win Pa Pa Tun, Banyar Maung Maung, Aung Than, Wanitda Watthanaworawit, Napaporn Kaewphanderm, Gornpan Gornsawun, Aung Pyae Phyo, François Nosten

**Affiliations:** 1 Shoklo Malaria Research Unit, Mahidol-Oxford Tropical Medicine Research Unit, Faculty of Tropical Medicine, Mahidol University, Maesot, 63110, Thailand; 2 Division of Infectious Diseases, Advanced Clinical Research Center, The Institute of Medical Science, University of Tokyo, Tokyo, Japan; 3 University College London, London, WC1E6BT, UK; 4 Centre for Tropical Medicine and Global Health, Nuffield Department of Medicine, University of Oxford, Oxford, UK

**Keywords:** Coinfection, COVID-19, Tuberculosis, Active screening, sanatoria

## Abstract

**Background:**

Tuberculosis (TB) is a chronic condition, with overlapping symptoms to those of coronavirus disease 2019 (COVID-19). There has been inconsistent evidence on whether TB is a predisposing factor for developing severe COVID-19. The aim of this report is to explore whether TB influences the severity of COVID-19.

**Methods:**

COVID-19 cases at two TB sanatoria on the Thailand-Myanmar border were reviewed. Demographic, clinical and laboratory data including TB treatment and co-morbidities, were analyzed. Characteristics and COVID-19 clinical outcomes were compared between two groups of patients: TB and those without TB (the caretakers and the medical personnel). Multivariable ordered logistic regression was conducted to compare the risk of severe COVID-19 between the two groups.

**Results:**

Between September 2021 and March 2022, 161 COVID-19 cases were diagnosed. Over half of the COVID-19 patients were infected with TB (n= 104, 64.6%), and the rest were not (n=57, 35.4%). The median (interquartile range) age was 48 (33.5-57.0) and 27 (23-33) years in the TB and in the non-TB COVID-19 patients, respectively. Before COVID-19 infection, 67.1% (106/158) of patients had received at least one dose of COVID-19 vaccine. The median cycle threshold value at diagnosis was not different between TB (18.5, IQR 16.1-32.3) and non-TB patients (18.8, 15.1-30.0). Fever, gastrointestinal symptoms and ageusia were more common in non-TB patients. Six patients (3.8%, 6/156) all from the TB group became severe of which five (3.2%, 5/156) required oxygen therapy. One TB patient died (1/104, 0.96%) of lung cancer. After adjustment for potential confounders, the final clinical severity was not different between the two groups (adjusted odds ratio 1.40, 95% confidence interval 0.16–12.39).

**Conclusions:**

TB was not associated with severe outcomes in the two TB sanatoria. The high uptake of COVID-19 vaccination and active screening could have impacted on disease progression and prevented unfavorable outcomes.

## Introduction

Tuberculosis (TB) is an important communicable disease in Myanmar and Thailand as both have been enlisted as high TB burden countries by the World Health Organization (WHO) ^1^. The efforts to control the disease along the Thailand-Myanmar border have been compromised by the coronavirus 2019 (COVID-19) pandemic ^2, 3^. This pandemic has reversed years of gains in global TB control activities, especially access to TB diagnosis and treatment ^1^. To control COVID-19 infection, many countries closed their borders since early 2020, restricting migration of people as well as trans-border TB control activities across the Thailand-Myanmar border.

It is well established that the severity of COVID-19 infection is often affected by certain comorbidities such as cardio-respiratory diseases, diabetes and conditions that decrease immune defenses ^4, 5^. Therefore, it can be expected that TB would represent a considerable comorbidity and a risk factor for severe COVID-19 disease ^6, 7^ due to the pre-existing lung damage and reduced antibody and T-cell responses to COVID-19 infection ^5, 8, 9^. A systematic review which included 146 patients coinfected with TB and COVID-19 from 18 countries found that the mortality from COVID-19 was 13% in the coinfected patients, compared with the global average of 6.6% ^10^.

The deleterious or aggravating effect of COVID-19 and TB disease co-infection was also explored in a meta-analysis ^6^ focusing on the clinical characteristics and disease course among survivors and deceased. The main finding was the significantly higher pooled odds ratios in the COVID-TB group as compared to the non-TB “control” group COVID-19 infected patients: 2.21 (95% CI: 1.80, 2.70) for death and 2.77 (95% CI: 1.33, 5.74) for severe COVID-19 disease. Another systematic review found that COVID-TB patients had higher risk of severity ^4, 11^ as well as higher mortality; 13% compared to 6.6% in people with COVID-19 alone (global mortality) ^4^. One study stated that coinfected patients had similar risk of mortality as well as prolong recovery times from COVID-19 ^12^ compared to non-TB COVID patients. Most of the studies in these two reviews were cases reports or case series including only COVID-TB coinfected patients without comparator or a few studies with non-TB control group either COVID-19 outpatients or those hospitalized for other morbidities. There are multiple studies ^13– 15^ concluding that coinfection did not cause statistically significant changes in mortality and disease severity of COVID-19 infection. A cross-sectional study stated that there may be an increased risk of contracting COVID-19 in patients who had a pre-existing TB diagnosis, but there was no statistically increased incidence of Intensive Care Unit admission, intubation or mortality rate when compared with those in the non-TB infected patients ^14^. However, patients in the non-TB comparator group were older-aged with co-morbidities which could be the main reason for being hospitalized and being more sick than younger COVID-TB co-infected patients.

**Figure 1.**
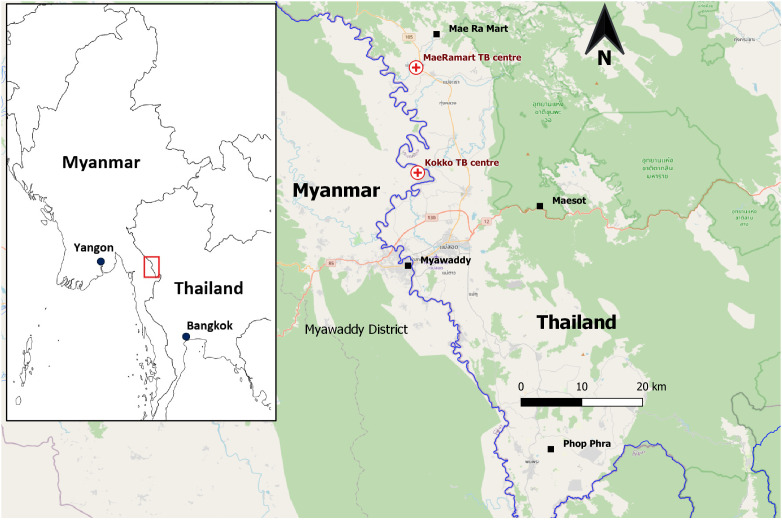
Geographical locations of SMRU two TB treatment sanatoria.

**Figure 2.**
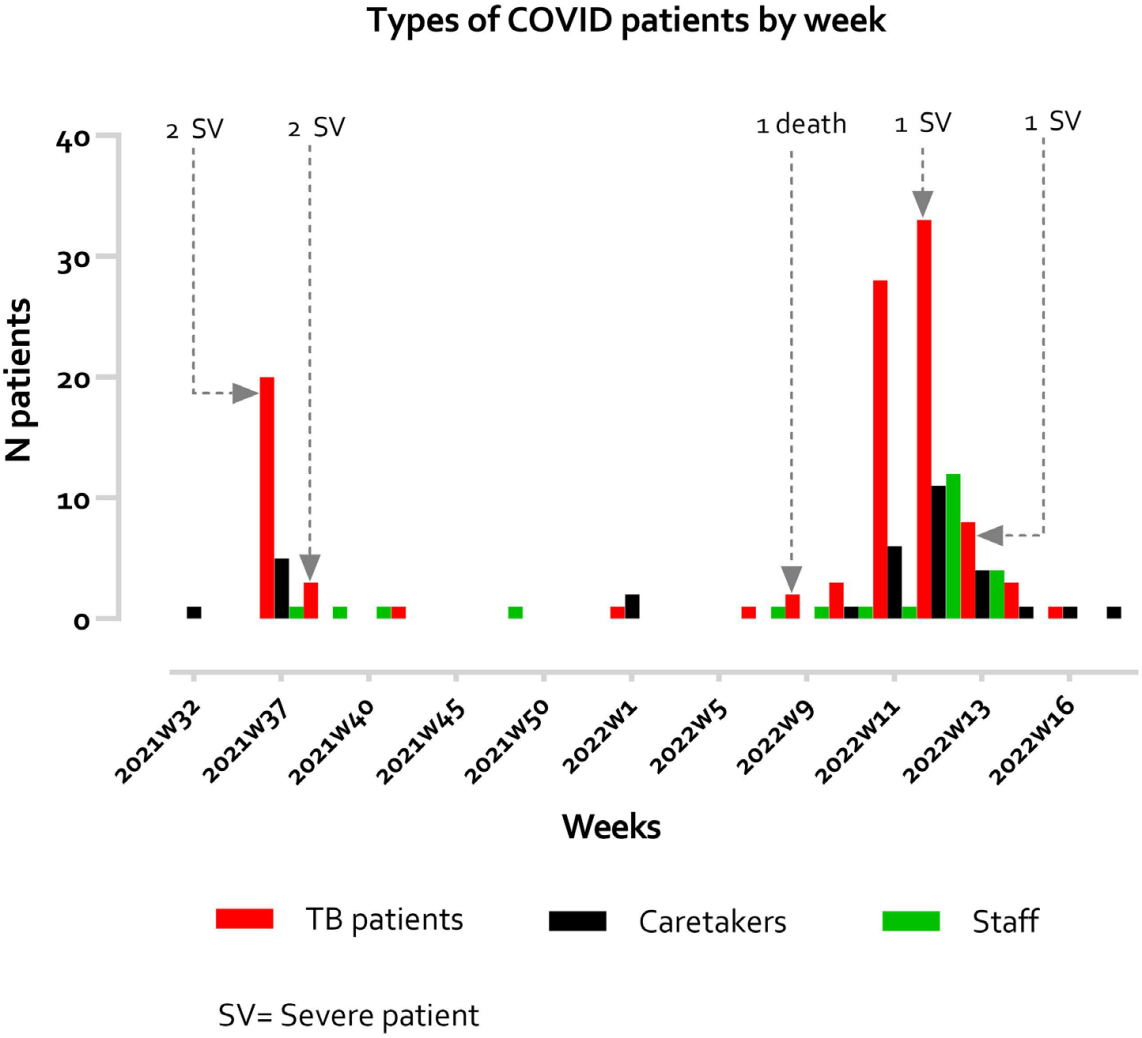
Types of COVID-19 patients by week.

Shoklo Malaria Research Unit (SMRU) is one of the health-service providing organizations on the Thailand-Myanmar border providing TB diagnosis, care and treatment and control activities ( Figure 1). Two TB treatment centers (one in Thailand and the other in Myanmar) adopting a sanitarium model were established in 2010. During the COVID-19 pandemic, two outbreaks of COVID-19 occurred in these two TB sanatoria involving both TB patients and people without TB (i.e., their caregivers in the compounds and medical staff). The aim of this report is to describe the two COVID-19 epidemics between September 2021 and April 2022 ( Figure 2). We compared the characteristics and outcomes of COVID-19 between TB and non-TB patient groups, as well as the association between TB infection and COVID-19 severity.

## Methods

### Study setting

Both TB sanatoria provide a residential care to all TB patients who are registered in the SMRU TB program. The program is specifically designed for underserved migrants, ethnic minorities and displaced populations along the border who have difficulties to access proper health care and in whom adherence to treatment is poor. Once patients are diagnosed with TB, they are treated free of charge with the WHO-recommended regimens along with the accommodation, nutrition and psychosocial supports throughout the course of treatment.

TB patients are accommodated in a single separated room, each having 10sqft in dimension and well designed for adequate airflow and lighting. The rooms are organized in a way to facilitate infection control and by types of TB, stages of anti-TB treatment and bacteriological clearance status (smear positive, smear negative and multi-drug resistant [MDR]-TB treatment areas). Strictly Directly Observed Treatment (DOT) is applied for anti-TB medications by the medical staff. The comorbidities are also treated by experienced medical teams in each center during the course of treatment. Family members of the patients are allowed to stay in the sanatoria and take care for their relatives.

### Study design and study population

This is a retrospective cohort analysis carried out on patients who were residing in two TB sanatoria during the pandemic period from September 2021 to April 2022, and who contracted COVID-19 infection. The study involved extracting and merging demographic, clinical, and diagnostic results from routinely collected patients charts, forms and laboratory database. The cohort comprised active TB patients, their caregivers, and medical staff, with the exclusion of one patient who was lost to follow-up and had unknown outcomes. 

### Diagnosis of TB and COVID-19

Tuberculosis was microbiologically diagnosed by using conventional microscopic examination (Ziehl-Neelsen stain), molecular technique (GeneXpert system) and radiologically by chest X-ray along with clinical examination. In case of rifampicin resistance detected by GeneXpert, anti-TB drug susceptibility testing was performed.

COVID-19 infection was diagnosed by taking nasopharyngeal swab and tested by either real-time reverse transcription polymerase chain reaction (RT-PCR), including Sansure ^®^ Novel Coronavirus (2019-nCoV) Nucleic Acid Diagnostic kit (detects N and ORF1ab genes) and/or Xpert Xpress SARS-CoV-2 ^16^ (detects E and N2 genes); or immunochromatographic antigen based rapid test (RDT), Standard Q COVID-19 Ag. All the diagnostics were performed according to the manufacturers’ instructions and guidelines from Ministry of Public Health, Thailand ^17^.

### Containment and control measures

Strict infection control measures against COVID-19 were applied in both TB treatment centers in accordance with the instructions released by the local and countries health authorities. Moreover, active screening of COVID-19 infection was applied to all the new coming TB patients and caretakers at the admission to the TB centres and whenever they were symptomatic or in contact with cases of COVID-19 confirmed cases within the treatment centers. All residences including medical staff were tested for COVID-19 infection if they were clinically suspected or in contact with confirmed patient of COVID-19. Movement restrictions amongst the patient buildings were also deployed by defining the infectious zones within the treatment centers. COVID-19 vaccines were provided in January 2021 in the treatment center in Thailand and then expanded to the treatment center in Myanmar in April 2021.

### Case definition and case management

The case definition and COVID-19 case management guidelines were based on the updated recommendations from by the Disease Control Department of the Ministry of Public Health, Thailand ^17^, WHO ^18– 20^ and the Centers for Disease Control and Prevention, United States ^21^.

At the time of diagnosis, COVID-19 cases were classified as:

- Asymptomatic COVID-19 confirmed case
- Confirmed case with mild symptoms and no risk factors
- Confirmed case with mild symptoms and risk factors
- Confirmed case with pneumonia and hypoxia

Risk factors included: age over 60 years old; chronic obstructive pulmonary disease (COPD) and other chronic lung diseases (excluding pulmonary TB); chronic kidney disease; chronic cardiovascular disease and congenital heart disease; cerebrovascular disease; diabetes; obesity (body mass index ≥ 35 kg/m ^2^); cirrhosis; immune deficiency conditions, and lymphocyte count < 1000 cells/mm ^3, 17^.

### Data collection

The demographic and clinical data of the patients were obtained from their case report forms. The data extraction process was performed by the investigators, who were also the clinicians responsible for the patients' care. The extraction involved collecting information from the patients' routine clinical records and registry books, which was then entered into an Excel spreadsheet. To ensure accuracy, the data was cross-checked by another clinician. Some information relating to vaccination and clinical details were missing as they were not recorded systematically. The laboratory information such as diagnosis and investigation results of individual patients were extracted from the laboratory database.

### Outcomes

The clinical severity of COVID-19 infection was categorized as follow:

- asymptomatic
- mild/moderate (symptomatic patients without evidence of pneumonia or hypoxia (or) clinical signs of pneumonia (RR>18/min) with S _P_O _2_ > 91% on air)
- severe (S _P_O _2_ < 90% on air (or) RR> 30/min) and critical (respiratory failure required mechanical ventilation, septic shock, or other organs failure requiring ICU care).

COVID-19 outcomes were categorized as fully recovered, recovered with sequels, death and loss to contact.

### Data analysis

A descriptive analysis was performed for all COVID-19 infected patients who resided in the TB sanatoria. The variables were summarized by percentage for categorical variables and mean and standard deviation or median with interquartile range for continuous variables. Outcomes of COVID-19 were compared between TB patients and people without TB. For normally distributed continuous variable, unpaired t-test or ANOVA were used. For those data which could not be converted into a standard distribution, Mann-Whitney U test was used. For categorical variables, Fisher’s exact test were used. For those with more than two categories, Bonferroni’s correction was used for assessing the statistical significance of the pairwise comparisons for each category. C-Reactive Protein (CRP) was grouped into four to reflect a level of severity of inflammation ^22, 23^. The clinical severity was compared between TB and non-TB COVID-19 patients using ordered logistic regression. The potential confounders (age, sex, vaccination status, and presence of comorbidity, cycle threshold [Ct] value at diagnosis, active case finding, and calendar year) were adjusted in the multivariable analyses. We used cluster-robust standard error accounting for the clustering within the two clinics. Proportional odds assumption was checked with *omodel* command in Stata MP 18.0 (StataCorp, TX, USA) and goodness of fit was assessed by ordinal version of the Hosmer–Lemeshow test using *ologitgof* command. Multiple imputation using chained equations was conducted for Ct value and vaccination status using *mi impute* command for 20 times and combined by Rubin’s rules ^24^ using *mi estimate* command. All confounders, the outcome and study site were included in the imputation model. We used logistic regression for vaccination status, ordered (ordinal) logistic regression for severity grade, and truncated regression for Ct values (limiting to 0 to 45) for imputation. The observations with missing outcome (n=6) were used for imputation but were excluded from the analysis models.

### Ethics approval

The analysis plan was presented to the local ethic advisory board of the Tak Province Border Community Ethics Advisory Board (T-CAB) ^25^, which provided for the use of routinely recorded patient records with anonymization of personal data.

The study synopsis was also presented to the Oxford Tropical Research Ethics Committee (OxTREC) and waiver was granted.

## Results

A total of 161 COVID-19 infected patients were diagnosed at two SMRU TB sanatoria during a period from 1 September 2021 to 30 April 2022. There were two outbreaks in September 2021 and March 2022 ( Figure 2), during which the Delta and Omicron variants circulated in this area, respectively. Some information relating to vaccination (in 2 TB and 4 non-TB patients) and clinical details (in 5 non-TB patients) were missing.

A summarized description on demographic, epidemiology and clinical information of all COVID-19 infected patients was described in Table 1.

**Table 1.** Demographic, epidemiological and clinical characteristics of 161 COVID-19 infected patients in two TB sanatoria on the Thailand-Myanmar border in 2021–2022.

	TB patients (n=104)	Non-TB patients * (n=57)	p-value
**Clinic**			
** TB sanatorium in Thailand**	37/104 (35.6)	40/57 (70.2)	<0.001
** TB sanatorium in Myanmar**	67/104 (64.4)	17/57 (29.8)	
Period			
** First outbreak (September 2021)**	24/104 (23.1%)	10/57 (17.5%)	0.55
** Second outbreak (March 2022)**	80/104(76.9%)	47/57 (82.5%)	
**Age (years)**	48 (33.5-57.0)	27 (23-33)	<0.001
**Male**	62/104 (59.6)	28/57 (49.1)	0.20
COVID-19 vaccination completion status at the time of COVID-19 infection			
** No vaccination**	37/103 (35.9)	15/55(27.3)	<0.001
** 1 ^st^ dose received**	29/103 (28.2)	5/55 (9.1)	
** 2 ^nd^ dose received**	37/103 (35.9)	21/55 (38.2)	
** Booster dose received**	0/103 (0)	14/55 (25.5)	
Type of COVID-19 vaccination (first dose)			<0.001
** Astrazeneca**	21/66 (31.8)	2/40 (5.0)	
** Covishield**	13/66 (19.7)	6/40 (15.0)	
** Sinopharm**	19/66 (28.8)	9/40 (22.5)	
** Sinovac**	13/66 (19.7)	23/40 (57.5)	
** Pfizer**	0/66 (0)	0/40 (0)	
Comorbidity			<0.001
** HIV**	13/104(12.5)	3/57(5.3)	0.18
** Diabetes**	9/104 (8.7)	1/57(1.8)	0.10
** Renal disease**	3/104 (2.9)	1/57(1.8)	1.00
** Asthma or obstructive lung diseases**	6/104 (5.8)	1/57(1.8)	0.42
** Hypertension or heart disease**	11/104 (10.6)	0/57(0)	0.01
Diagnosis tools of COVID-19 infection			
** Reverse Transcription PCR**	87/104 (83.7)	53/57 (93.0)	0.14
** Rapid Diagnostic Test**	17/104 (16.4)	4/57 (7.0)	
Type of TB diagnosed			
** Sputum-positive pulmonary TB**	65/104 (62.5)	NA	NA
** Sputum-negative pulmonary TB**	35/104 (33.7)	NA	
** Extra-pulmonary TB**	4/104 (3.9)	NA	
Type of TB treatment			
** Initial treatment regimen (IR)**	68/104 (65.4)	NA	NA
** Retreatment regimen (RR)**	21/104 (20.2)	NA	
** Multi-Drug Resistant regimen (MDR-TB)**	15/104 (14.4)	NA	
Timing of TB and COVID-19 diagnosis			
** TB diagnosed before COVID-19 infection**	102/104 (98.1)	NA	NA
** TB diagnosed after COVID-19 infection**	2/104 (1.9)	NA	
** Days to COVID-19 diagnosed following TB treatment**	110 (53-167)	NA	
** Days to TB treatment following COVID-19 infection**	28 (7-50)	NA	

*Non-TB patients include caretakers (33) and health care workers from two TB sanatoria (24). NA: not applicable Proportion or median (Inter-quartile range) is shown. P-values were derived by either Fisher’s exact test or Mann-Whitney’s U test.

Over half of the cohort were TB patients (n= 104, 64.6%). The second group was non-TB infected patients (n=57, 35.4%), with caretakers to TB patients (33/57, 57.9%) and health care workers of two TB sanatoria (24/57, 42.1%).

TB patients were more likely to have at least one comorbidity (39/104, 38.0%) than non-TB patients (5/57, 8.8%) (p<0.001). Amongst comorbidities, HIV-coinfection was the most common (n=16/161, 9.9%) followed by diabetes (10/161, 6.2%), hypertension (8/161, 5.0%), chronic respiratory diseases (6/161, 3.7%), and renal disease (1/161, 0.6%). A total of six TB patients and one non-TB patient had multiple comorbidities. Overall, 67.1% (106/158) received at least one dose of a COVID-19 vaccine at least 14 days before they were tested positive for COVID-19: 64.1% (66/103) in TB group and 72.7% (40/55) in non-TB group. Two or more doses of vaccination was given to 35.9% (37/103) of the TB group and 63.6% (35/55) of the non-TB group.

Amongst the COVID-19-infected TB patients, 98.1% (102/104) had already started treatment for TB at the time of diagnosis of COVID-19 whereas remaining two patients (1.9%) were diagnosed as TB after being infected with COVID-19. The median (IQR) time of COVID-19 diagnosis from the start of TB treatment among them (n=102) was 110 days (53–167).

### Description of COVID-19 infection

The details of clinical presentation, outcomes and laboratory results were summarized in Table 2. 

**Table 2.** Descriptive analysis of COVID-19 infected patients who resided in two TB sanatoria. N (%).

	TB patients (n=104)	Non-TB patients (n=57)	p-value
**Active screening**	72/104 (69.2)	27/57 (47.4)	**0.006**
**Ct value (SARS2 N gene) at diagnosis**	18.5 (16.1-32.3)	18.8 (15.1-30.0)	0.47
Signs and symptoms of COVID-19 infection	(n=156)		
** Fever**	38/101 (37.6)	37/55 (67.3)	<0.0001
** Respiratory system**	80/101 (79.2)	43/55 (78.2)	0.88
** Gastrointestinal system**	2/101 (2.0)	6/55 (11.1)	0.02
** Musculoskeletal system**	50/101 (49.5)	30/55 (54.6)	0.55
** Loss of smell**	10/101 (9.9)	7/55 (12.7)	0.60
** Loss of taste**	24/101 (23.8)	24/55 (43.6)	0.01
Case classification at time of COVID-19 diagnosis (n=157)			<0.001
** Asymptomatic**	11/102 (10.8)	9/55 (16.4)	
** Mild symptoms with no risk factors**	49/102 (48.0)	41/55 (74.6)	
** Mild symptoms with risk factors**	39/102 (38.2)	5/55 (9.1)	
** Symptomatic with severe pneumonia**	3/102 (2.9)	0/55 (0)	
Worst clinical severity during the course of COVID infection (n=155)			0.64
**Asymptomatic**	17/100 (17.0)	10/55 (18.2)	
**Mild/Moderate**	77/100 (77.0)	44/55 (78.2)	
**Severe**	6/100 (6.0)	1/55 (3.6)	
Laboratory investigation at baseline			
** Conducted**	58/101 (57.4)	6/55 (10.9)	
** Abnormal Complete Blood Count**	33/58 (56.9)	1/6 (16.7)	<0.001
** C-Reactive Protein grading (n=60)**			1.00
** Normal (<8 mg/L)**	30/55 (54.6)	3/5 (60.0)	
** Grade I (8-20mg/L)**	11/55 (20.0)	1/5 (20.0)	
** Grade II (21-40mg/L)**	7/55 (12.7)	1/5 (20.0)	
** Grade III (>40mg/L)**	7/55 (12.7)	0/5 (0)	
Treatment (n=156)			
** Antibiotics**	13/101 (12.9)	3/55 (5.5)	
** Anticoagulant**	2/101 (2.0)	3/55 (5.5)	
** Antiviral**	10/101 (9.9)	1/55 (1.8)	
** Systemic steroid**	6/101 (5.9)	2/55 (3.5)	
** Oxygen therapy**	5/101 (5.0)	0/55 (0)	
Outcomes of COVID-19 infection (n=161)			0.58
** Fully recovered**	103/104 (99.0)	56/57 (99.0)	
** Recovered with sequels**	0/104 (0)	0/57 (0)	
** Death**	1/104 (1.0)	0/57 (0)	
** Loss to contact**	0/104 (0)	1 */57 (1.0)	

P-values were derived by either Fisher’s exact test or Mann-Whitney’s U test. *One COVID-19 positive case from non-TB patient group (caretaker to TB patient) has left for home inside Myanmar. She was well on last contact with the clinic a week after abscondment.

Over 87% of cases were diagnosed with RT-PCR test (140/161). Among the diagnosed cases of COVID-19 by RT-PCR, the median (IQR) of Ct value of SARS2 N gene was 18.5 (16.1-32.3) in TB patients (n=104), compared to 18.8 (15.1-30.0) in non-TB infected COVID-19 patients (n=57). There was no significant difference in Ct values between the two groups. 

At the time of diagnosis, the most common clinical presentations were cough with sneezing (78.9%, 123/156), body ache and pain (51.3%, 80/156), fever (48.1%, 75/156) and impaired taste function (30.8%, 48/156). Some clinical symptoms were significantly more common in non-TB patient group, particularly fever (p<0.0001), gastrointestinal symptoms (p 0.02) and impaired taste function (p 0.01). Clinical information for four patients were missing or incomplete and were dropped for analysis.

In terms of clinical severity, most of the COVID-19 cases (121/155, 78.1%) were defined as “mild to moderate”, a few cases (7/155, 3.9 %) were “severe” because of S _P_O _2_<90% on ambient air or respiratory rate (RR) > 30/min and the rest (27/155, 17.4%) were “asymptomatic”. Systemic steroid was used in 8 out of 156 (5%) patients, and oxygen therapy with nasal cannula was needed during the course of infection for 5 out of 156 (3%) cases. All the cases that required oxygen were in the TB-infected group. Among them, one TB patient had to take low dose oxygen therapy for the underlying COPD before he was diagnosed with COVID-19 infection. Among patients with severe symptoms six were TB patients and one was clinic staff. Of the six TB cases two were on initial TB treatment, three on retreatment, one was on MDR regimen. Three patients with TB had oxygen drop (S _P_O _2_<94%), three had moderate anaemia and one had mild anaemia ^26^. Two TB patients had co-morbidity; one with HIV infection and the other with mental disorder.

After adjusting for age, sex, vaccination status, presence of comorbidity, Ct value at the diagnosis, diagnosis by active case finding, and calendar year, the risk of developing severe disease of COVID-19 was not different in TB-patients compared with non-TB patients (adjusted odds ratio 1.40, 95% CI 0.16-12.39, p=0.76). Younger age, female sex, higher Ct value at baseline, diagnosis by active case finding, and infections in 2022 (assumed Omicron variant) compared with those in 2021 (assumed Delta variant) was associated with lower risk of developing severe disease ( Table 3).

**Table 3.** Ordered logistic regression analysis for the odds of higher clinical severity of COVID-19 in TB and non-TB patients.

	N	Univariable	Multivariable (CC)	Multivariable (MI)
Characteristic		OR (95% CI)	OR (95% CI)	OR (95% CI)
TB patients	155	1.30 (0.09-18.22)	1.46 (0.20-10.50)	1.40 (0.16-12.39)
Age (year)	155	1.02 (0.97-1.06)	1.01 (1.01-1.01)	1.01 (1.00-1.02)
Male	155	1.56 (0.93-2.61)	2.03 (1.54-2.66)	1.88 (1.17-3.03)
Vaccination status	152			
0 or 1		Reference	Reference	Reference
2 or 3		0.90 (0.17-4.84)	1.81 (0.52-6.36)	1.41 (0.54-3.64)
Comorbidity	155	1.40 (0.85-2.32)	1.08 (0.73-1.61)	0.93 (0.55-1.57)
CT value at diagnosis	136	0.95 (0.89-1.01)	0.95 (0.90-1.00)	0.96 (0.90-1.00)
Omicron (Year 2022)	155	0.39 (0.21-0.73)	0.21 (0.13-0.33)	0.25 (0.22-0.29)
Active case finding	155	0.28 (0.08-1.01)	0.36 (0.14-0.93)	0.29 (0.16-0.53)

CC: Complete case analysis (n=133), CI: confidence interval, MI: multiple imputation analysis, N: number assessed, OR: Odds ratio. Clustered sandwich estimator is used for clustering within two clinics. Goodness-of-fit Hosmer-Lemeshow test: p=0.68, Proportional odds assumption test: p=0.93.

A total of 159 out of 161 patients (98.8%) fully recovered from COVID-19 infection but one patient (1/104, 0.96%) in TB-infected group died. The overall mortality in this cohort was 0.62% (1/161). This was a 73-year old man with underlying sputum smear negative pulmonary TB who received a retreatment regimen due to presumptive TB clinical symptoms and radiological findings on chest X-ray. Sputum microscopy smear examination and molecular testing for sputum specimens were negative. The radiological finding showed a malignant mass in upper and middle zone of the right lung with adjacent structural invasion and ribs destruction. He received Astra-Zeneca vaccine two weeks after TB treatment started. He had significant weight loss with no clinical progress during two-month initial phase of TB treatment and got infected with COVID-19. He was treated with parenteral antibiotics, anticoagulant, systemic steroids and other palliative care but the deterioration was progressed to death. This patient probably died of underlying pulmonary malignancy compounded by COVID-19 infection.

## Discussion

Reflecting worldwide epidemic waves of the Delta and Omicron variants of COVID-19 infection, two outbreaks of COVID-19 infection occurred at the SMRU TB sanatoria on Thailand-Myanmar borders between September 2021 and March 2022.

Respiratory (sneezing, cough, and sore throat) and musculoskeletal (joint pain and muscle ache) symptoms were the most common presentation of COVID-19 infection in the cohort. However, fever (p<0.0001), gastrointestinal related symptoms (p=0.02) and impaired sensation on taste (hyposmia to ageusia) (p=0.01) were more common in the non-TB patient group. In the TB patient’s cohort, having complex symptoms of TB disease and side effects of anti-TB medicines could mask the typical presentation of COVID-19 infection compared to relatively healthy group of non-TB patients.

There were no major differences in disease severity and outcomes of COVID-19 infection between the TB and non-TB groups after adjustment for potential confounders. In terms of case management, there was no case requiring the transfer to intensive care during the course of COVID-19 infection. There was one fatality in a COVID-19/TB co-infected elderly man, probably due to lung cancer. The overall situation was comparably less severe than the morbidity and mortality reported in local and regional COVID-19 statistics. The findings from this report are similar to other studies showing that COVID-19/TB co-infection does not cause statistically significant increases in mortality or disease severity ^13, 15^. Moreover, COVID-19/TB coinfection may result in a less severe presentation and course of infection ^14^, particularly through active case finding (i.e. earlier detection) for COVID-19 was taken place in TB patients.

This is contrary with the findings from other studies showing that tuberculosis represents a considerable comorbidity and risk factor for severe COVID-19 disease ^5, 11^. However, one must be cautious in interpreting findings from the studies because they may not widely reflect the general population, and the studied group were potentially hospitalized as a precautionary measure due to their pre-existing diagnosis of tuberculosis which could have introduce a bias in comparing the COVID-19 only group, who were admitted due to clinical severity of their infection. The authors acknowledged that the finding may be cofounded by multiple factors including epidemiological disparities between the groups ^14^. In this cohort, although non-TB cases were younger and had less comorbidities than TB-infected cases, the clinical severity and overall outcome was not different between TB and non-TB patients, which could be due to the active screening of TB patients who, as a result, were diagnosed earlier. On the other hand, non-TB patients included in this study were not selected based on clinical severity or the risks of developing severe COVID-19 disease. Although the number of non-TB patients was small, this group can be more representative of the general population in this area, which was not the case in most other hospital-based studies. Earlier detection of COVID-19 infection because of active screening at the entry and higher uptake of COVID-19 vaccine in this cohort and predominance (79%, 127/161) of infections in 2022 (presumably Omicron variant) might have been one reason for the overall good clinical outcomes in both TB-infected and non-infected patients.

There were some limitations in the study. Although we compared the clinical severity adjusted by potential confounders with multiple imputation for missing variables to take into account of the observational nature of this cohort, there could be unobserved residual differences between TB and non-TB patients. Particularly, modifications in case classification, severity level and treatment guidelines from time to time has given inconsistency at individual patient management. As the resources were limited, a systemic and continuous viral load monitoring using molecular testing could not apply to detect a definite negativity of COVID-19 infection in every cases. Although the overall severity was not so high and was not very different between TB and non-TB patients, only one mortality in this cohort was a TB-infected patient and our relatively small number of patients did not allow us to assess whether there was a clear difference in mortality between TB and non-TB patients. Hence, the results should be interpreted with caution.

Although the published literature on TB/COVID-19 coinfection has increased in recent times, there appears to be little consensus to the extent to which TB is a risk factor for severe COVID-19 infection. The presence of confounding variables that may influence the above findings and highlight the need for careful factor analysis to ascertain which conclusions are accurate and clinically applicable. It is an important area of continued research as the elucidation of the pathophysiological mechanisms surrounding coinfection will be a vital influence on public health initiatives, infection control protocols and shielding guidance for patients who are suffering from TB in times when COVID-19 prevalence rates are still a cause for global concern.

## Data Availability

Raw data and related database are available on request according to the MORU Tropical Health Network data sharing policy. The minimal dataset, devoid of patient confidential data, has been deposited at Zenodo under the DOI 10.5281/zenodo.10042760. Full dataset can be applied for via the MORU website or from MORU data sharing committee datasharing@tropmedres.ac. Zenodo: Minimal dataset for the research "Outbreaks of COVID-19 in a tuberculosis treatment sanatorium on the Thailand-Myanmar border: a retrospective cohort analysis". https://doi.org/10.5281/zenodo.10042760 ^27^. This project contains the following underlying data: Data file 1. COVID_TB_Data for sharing.dta - Individual patient data devoid of confidential information. Data file 2. Outbreaks of COVID-19 in a tuberculosis treatment sanatorium on the Thailand-Myanmar border.do - STATA .do file of the analysis. Data are available under the terms of the Creative Commons Attribution 4.0 International license (CC-BY 4.0).
