# Supplementary material for: Outbreaks of COVID-19 in a tuberculosis treatment sanatorium on the Thailand-Myanmar border: a retrospective cohort analysis
Source: Wellcome Open Res. 2023 Nov 1;8:272. Originally published 2023 Jun 22. [Version 2] doi: 10.12688/wellcomeopenres.19275.2 (PMC11600724; doi:10.12688/wellcomeopenres.19275.2)
Supplement: Peer review history

# Reviewer report: Reviewer response for version 2

**DOI:** 10.21956/wellcomeopenres.22544.r106859

**Published:** 2024-11-26

**Authors:** Karikalan Nagarajan

**Affiliations:** 1 ICMR-National Institute for Research in Tuberculosis (NIRT), Chennai, Tamil Nadu, India

This study uses a retrospective cohort analysis for understanding  TB as a predisposing factor for developing severe COVID-19. 

 But as the authors themselves had mentioned that differences in severity level and treatment guidelines for Covid/TB  have lead to  inconsistencies. This would be a serious limitation of this study which could impact the findings. Without a more detailed sub-grouping and reanalysis taking into account of such clinical factors the paper findings may not relevant. 

 Second, there are other com morbidities like diabetes which severity impact Covid-19 manifestation and progress . Such factor may confound the analysis with respect to TB impact on Covid-19. The authors have not given any details how the matching was done with respect to such risk factors. 

 Mention sample size  justification , case -control ratio allocation,  time line of retro inclusion of patients  and other considerations. 

 Comparability of  TB sanatorium services needs to mentioned with respect to patient load, type of patients cared and treatment management practices etc. 

Is the work clearly and accurately presented and does it cite the current literature?

Partly

If applicable, is the statistical analysis and its interpretation appropriate?

Partly

Are all the source data underlying the results available to ensure full reproducibility?

Yes

Is the study design appropriate and is the work technically sound?

Yes

Are the conclusions drawn adequately supported by the results?

Partly

Are sufficient details of methods and analysis provided to allow replication by others?

Partly

Reviewer Expertise:

Public health, TB, Covid-19 research

I confirm that I have read this submission and believe that I have an appropriate level of expertise to confirm that it is of an acceptable scientific standard, however I have significant reservations, as outlined above.

# Reviewer report: Reviewer response for version 2

**DOI:** 10.21956/wellcomeopenres.22544.r87445

**Published:** 2024-07-30

**Authors:** Lucia Cilloni

**Affiliations:** 1 Johns Hopkins Bloomberg School of Public Health, Baltimore, MD, USA

The article conducts a retrospective analysis of clinical and lab data from two TB sanatoria in Thailand and Myanmar to assess whether TB patients experience worse COVID-19 severity compared to caretakers and medical staff at the facilities, during two outbreaks in 2021 and 2022. The authors present the demographic and clinical patient data and conduct regression analysis to determine the presence of significant differences between the TB and non-TB individuals who had COVID-19. The authors found no significant differences in the disease severity between the two groups. They did observe significant differences in the presentation of COVID-19 disease (fever and loss of taste were more common in the non-TB group). 

 The data and the analyses are well-presented and clear. The authors highlight some limitations to their study that may have biased the lack of evidence that TB may increase the severity of COVID-19 disease, which has been documented by other studies. Reference 11 is used as an example of studies that found that TB was a comorbidity for more severe COVID-19, but that article highlights their findings were not significant, which should be noted.

 A potential bias that isn't highlighted by the authors of this paper is the fact that almost all of the individuals in the TB group had already been on TB treatment for approximately 3 months, which may influence how much more susceptible these individuals may be to COVID-19 compared to individuals without TB. It may be interesting to see if severity differed by treatment initiation date, although I appreciate there would be an important data limitation for this analysis.

 I was also wondering why there was such a striking difference in the number of COVID-19 cases between the two groups in the two sanatoria- in Thailand over 70% of staff got COVID-19 compared to just 29% in Myanmar. Were there demographic or clinical differences between the staff (non-TB individuals) of the two structures?

 Finally, for the one individual that passed, it may be appropriate to discuss their demographic and clinical characteristics, to get a better picture of them, since they do get highlighted at the end because they belong to the TB group.

Is the work clearly and accurately presented and does it cite the current literature?

Yes

If applicable, is the statistical analysis and its interpretation appropriate?

Yes

Are all the source data underlying the results available to ensure full reproducibility?

Yes

Is the study design appropriate and is the work technically sound?

Yes

Are the conclusions drawn adequately supported by the results?

Yes

Are sufficient details of methods and analysis provided to allow replication by others?

Yes

Reviewer Expertise:

Mathematical modelling, statistics, epidemiology, TB, COVID-19

I confirm that I have read this submission and believe that I have an appropriate level of expertise to confirm that it is of an acceptable scientific standard.

# Reviewer report: Reviewer response for version 2

**DOI:** 10.21956/wellcomeopenres.22544.r69515

**Published:** 2024-05-25

**Authors:** Marc Y R Henrion

**Affiliations:** 1 Statistical Support Unit, Malawi Liverpool Wellcome Programme, Blantyre, Malawi; 2 Department of Clinical Sciences, Liverpool School of Tropical Medicine, Liverpool, London, UK

SUMMARY OF REVIEW

 I thank the authors for addressing many of my previous comments, and particularly for making the minimal data and the analysis code available now.

 However, while most were addressed, some of my previous comments remain unchanged and I had a few additional comments to the revised manuscript. There is, however, only one major comment I would still like to see addressed, the others are not major, and I leave these to the discretion of the authors to revise their article.

 MAJOR COMMENT:

 In the discussion, the authors state:

 "There were no major differences in disease severity and out- comes of COVID-19 infection between the TB and non-TB groups after adjustment for potential confounders. [...] This is contrary with the findings from other studies showing that tuberculosis represents a considerable comorbidity and risk factor for severe COVID-19 disease."

 As per comment A6 below (and made in my previous review, comment S4 from that review, but admittedly not clearly or strongly enough), this is not a correct interpretation. The authors mean to say there they did not find a statistically significant difference in severity between TB and non-TB patients. The direction of effect (adjusted OR from multiple imputation analysis 1.40; i.e. increased odds of a more severe outcome in the TB patients) is very much in line with the studies that have showed comorbidity of COVID-19 and TB to be a risk factor - so it is wrong to state that the presented data contradict previous findings. They agree, even if in the present study this effect was not shown to be statistically significant.

 I urge the authors to rephrase their interpretation accordingly.

 STATISTICAL METHODS COMMENTS

 COMMENT S1: Thanks for making the minimal data and Stata do file available. For the multiple imputation, I think the methods section should still be a bit clearer. From the Stats code, as far as I can tell (I am not a regular Stata user), a mix of regression models from ordered logistic regression, logistic regression and truncated regression was used for the different variables. Might be good to clearer to spell this out in the methods section - but admittedly the sharing of the analysis code makes this unambiguously clear now.

 ANALYSIS & RESULTS MINOR COMMENTS

 COMMENT A1: The authors state in the methods section that we categorical variables with more than 2 levels, Bonferroni correction was used to adjust p-values from pair-wise tests. For such variables, an omnibus test using either the Fisher or Chi-squared test should still be conducted, and pair-wise tests only done if the overall p-value is significant.

 COMMENT A2: The authors state that 6 records with missing outcomes were included in the imputation but excluded from the analysis. There is no reasons why these would need excluding from the analysis of the multiply-imputed data.

 COMMENT A3: Tables 1 & 2 still do not consistently list the percentage of missing data. This can be inferred by the reader and Table 2 lists the number of available records (given as "n=XX" after each variable name / description) for some of the variables (but strangely not all) but would be much clearer if this is stated clearly for each variable in each of the 2 tables. Table 3 does this quite nicely.

 COMMENT A4: Table 2, for the comparison of signs / symptoms, there should be an overall test for difference rather than the multiple tests for each specific sign (the latter is OK but the former should be done first).

 COMMENT A5: Table 2, unclear why treatment was not compared between the 2 groups (or maybe just an omission to include the p-value for that variable?).

 COMMENT A6: (See also the one major comment.) The way that non-significant associations are interpreted is still not fully correct. E.g. for the main outcome analysis, the authors state "After adjusting for age, sex, vaccination status, presence of comorbidity, Ct value at the diagnosis, diagnosis by active case finding, and calendar year, the risk of developing severe disease of COVID-19 was not different in TB-patients com- pared with non-TB patients (adjusted odds ratio 1.40, 95% CI 0.16-12.39, p=0.76)." But an OR of 1.40 hardly counts as "not different". What the authors mean is that the observed OR was not statistically significantly different from 1. So, the null hypothesis of no difference between the groups cannot be rejected, but that is not the same thing as saying that the 2 groups are the same / not different. Please amend. There are other similar incorrect interpretations (e.g. in the abstract "The median cycle threshold value at diagnosis was not different between TB (18.5, IQR 16.1-32.3) and non- TB patients (18.8, 15.1-30.0)." -- 18.5 is not equal to 18.8). All that is required in these instances is to change "not different" to "not statistically significantly different". For the discussion section, for the main analysis result, this needs a bit more rephrasing (it is wrong to state that these data contradict previous research if the direction of effect is the same), hence why I have listed that as a separate major comment.

 OTHER MINOR COMMENTS

 COMMENT M1: P-value reporting still inconsistently done to 2 or 3 digits. E.g. most exact p-values are given to 2 digits, but for p-values lower than 0.01, it seems that 3 digits are being used. Please use consistently 2 (and hence "<0.01" for low p-values) or 3 digits.

 TYPOS

 COMMENT T1: p. 6/22 (pdf download), data analysis section, 1st paragraph, line 7: "normally distributed variable"; this should be plural "normally distributed variables"

 COMMENT T2: p 10/22 (pdf download), 2nd column, 3rd paragraph: "one patient (1/104, 0.96%) in TB-infected group"; there is a missing definite article: "one patient (1/104, 0.96%) in the TB-infected group"

Is the work clearly and accurately presented and does it cite the current literature?

Yes

If applicable, is the statistical analysis and its interpretation appropriate?

Partly

Are all the source data underlying the results available to ensure full reproducibility?

Partly

Is the study design appropriate and is the work technically sound?

Yes

Are the conclusions drawn adequately supported by the results?

Yes

Are sufficient details of methods and analysis provided to allow replication by others?

Partly

Reviewer Expertise:

I am a biostatistician working mostly in infectious, tropical disease applications, including TB and SARS-CoV-2 / COVID-19 research.

I confirm that I have read this submission and believe that I have an appropriate level of expertise to confirm that it is of an acceptable scientific standard, however I have significant reservations, as outlined above.

# Reviewer report: Reviewer response for version 1

**DOI:** 10.21956/wellcomeopenres.21358.r64440

**Published:** 2023-08-18

**Authors:** Marc Y R Henrion

**Affiliations:** 1 Statistical Support Unit, Malawi Liverpool Wellcome Programme, Blantyre, Malawi; 2 Department of Clinical Sciences, Liverpool School of Tropical Medicine, Liverpool, London, UK

SUMMARY OF STUDY

 Htet Ko Ko Aung and colleagues present a retrospective cohort study conducted over the span of 8 months during 2 COVID-19 outbreaks in 2 tuberculosis (TB) clinics on the Thailand-Myanmar border. Participants include both TB patients at the 2 clinics as well as medical staff and caregivers (participants without TB).

 The authors describe demographic and clinical characteristics as well as clinical outcomes of study participants, stratifying by participant type (TB or no TB). The main analysis focuses on comparing disease severity (one of 3 levels: asymptomatic, mild/moderate and severe) between participant types. The main study conclusion is that there was no evidence in this study of differences in clinical severity of CVODI-19 between TB patients and participants without TB.

 I generally congratulate the authors on an informative and important study, employing generally sound scientific research methods. I do have some comments / queries which I give below in a structured format. Every comment is flagged by "COMMENT X:" where X is a number which hopefully makes it easier for the authors to respond to specific comments.

PRESENTATION

 Generally the manuscript is clear and accurate, with good referencing to other literature in the field. There are a number of minor typos and language issues that should be revised (see list below).

STUDY DESIGN

 The retrospective cohort design is generally appropriate to answer the primary research question (does COVID-19 severity differ between TB patients and people without TB). The specialised setting within TB clinics, with the control group consisting of caregivers and medical staff (i.e. individuals probably better informed about infection control, importance of vaccine and access to COVID-19 treatments) will however limit some of the generalisability of the study findings. Nevertheless, these findings are valid and important to share with the research community.

METHODS & REPRODUCIBILITY

 Neither the underlying data nor the analysis code are directly available.

 Data can be requested through the researchers' institution, but the link to do so only refer to the data sharing policy and not directly to instructions how to request this particular dataset.

 COMMENT R1: I would urge the authors to deposit their Stata analysis code on a code repository for easier reproducibility and removing any ambiguities about the specific analysis procedures that can be left from a text description of what was done.

 COMMENT R2: I would similarly urge the authors to deposit the minimal dataset needed to reproduce the analyses from the present manuscript on a data repository. I understand that this may not be possible due to data policies and local regulations, in which case the authors could consider deriving a synthetic dataset based on the real analysis dataset. At the very least a direct link to request access for this particular dataset should be provided - the fewer hoops to jump through, the better.

STATISTICAL ANALYSIS & INTERPRETATION

 Mostly the methods employed are clearly described, straightforward and appropriate. I do, however, have a few specific comments on the statistical analysis.

 COMMENT S1: Please provide full details of the ordinal logistic regression model that was used. I also did not see any discussion of model fit and diagnostics, but this is important given that ordinal logistic regression makes an assumption of proportional odds which should be checked.

 COMMENT S2: While participants are recruited from 2 different TB clinics, and Table 1 shows that these 2 clinics differ at least in the TB patient : non-tb participant ratio, the analyses do not seem to account for clinic. Presumably there may be all kinds of other differences, not captured through measured variables, that may impact on outcomes, so I would suggest that the authors adjust for clinic in the analysis model or at least do a sensitivity analysis to investigate the effect of clinic on the study conclusions.

 COMMENT S3: The authors make use of multiple imputation for the main analysis. This is great, but they should state clearly what imputation model was used with the MICE approach (presumably the Stata default, which would be PMM - but should be stated explicitly).

 COMMENT S4: The authors use a few times language that rules out an association or effect or difference (e.g. "TB was not associated with severe outcome" for the main study conclusion, "no difference between the two groups" for duration from vaccination to test positivity, "18.5 [...] which was not different from 18.8 [...]", ...). I think this language should be rephrased to make it clear that in this particular study there was not enough evidence to support such associations etc or that differences (e.g. 18.5 is not the same as 18.8; similar but not the same, so different) were not statistically significant. You do not have enough evidence to reject the null hypothesis in these cases, but that does not necessarily mean that the null is true.

MINOR COMMENTS

 COMMENT M1:  The study involved 161 participants. But some variables seem to have missing data (e.g. 133 is the number of participants included in the complete case analysis, 155 is the denominator for COVID-19 vaccination status, 155 (though this is given as 156 in the abstract) for disease severity, ...), it would be helpful if Tables 1 and 2 could also list the amount of missing data for each variable.

 COMMENT M2:  In the third paragraph of the introduction, the sentence "The main finding was the similar pooled odds ratios in the COVID-TB group as compared to the non-TB “control” group COVID-19 infected patients: 2.21 (95% CI: 1.80, 2.70) for death and 2.77 (95% CI: 1.33, 5.74) for severe COVID-19 disease." is not fully clear. At least specify what groups the OR quoted here compares - since the confidence intervals exclude 1, one could read this to mean that TB very much increases the risk of death or severe disease, but clearly that is not what the authors meant to say here.

 COMMENT M3: In the 'Containment and control measure' section, if I read it correctly then controls got tested daily for COVID-19 as they reported to the clinic but TB patients were only tested upon admittance? OR does 'active screening' mean continuous testing during the stay in the clinic. Maybe good to clarify how often TB and non-TB participants were tested for COVID-19.

 COMMENT M4: In the 'Data analysis' section, specify when the Chi-squared and when the Fisher test was used and/or indicate in a footnote to Tables 1 and 2 which test was used when.

 COMMENT M5: Please give a reference for Rubin's rules in the 'Data analysis' section.

 COMMENT M6: For Tables 1 and 2, it would also be great to see results stratified by clinic -- maybe as supplementary material tables?

 COMMENT M7: Table 1 and third paragraph of the results section: might be worth to also state the overall p-value for comparing co-morbidity between TB and non-TB participants (Table 1 only formally compares each specific co-morbidity while the text states any comorbidity but does not give a p-value). I am not too fussed about getting a p-value but given that p-values are calculated for most other things, it would be consistent to give one here too.

 COMMENT M8: Table 1, unclear why no comparison (p-value given as "NA") is made between TB and non-TB participants in distribution of first dose of COVID-19 vaccine.

 COMMENT M9: Last sentence of the third paragraph of the 'Results' section reads "Only 2 out of the 104 TB patients (1.9%) were diagnosed as TB after COVID-19". This is a bit puzzling as by design the TB patients would have been recruited based on their TB status (as patients of the clinics). Presumably either some patients had their TB identified as a result of showing up to a test centre for COVID-19 then got admitted or else some of the caregivers or medical staff became TB positive during the study. Might be worth clarifying what happened here.

 COMMENT M10: Table 2 for case classification at COVDI-19 diagnosis: unclear why each level is compared between groups rather than comparing the entire distribution of diagnosis levels (as done for other categorical variables with mutually exclusive levels in the very same table). I would recommend just giving a single p-value for comparing all levels between the 2 groups.

 COMMENT M11: p-values are variable reported to 2 or 3 decimal digits. Please give them consistently to the same number of decimal digits.

 COMMENT M12: I would probably put the case classification at COVID-19 diagnosis and the worst clinical severity during the disease course right next to each other in the table rather than having other variables summarised between them.

 COMMENT M13: Can you double-check the Ors for the vaccination status levels? While the CIs are large and include 1, the ORs are consistently >1, which would mean that 1 or 2+ doses of the vaccine had on average more severe COVID-19 disease than no vaccination (even if not statistically significant). This direction of effect seems puzzling. It may be true, but worth double-checking.

 COMMENT M14: Second last paragraph of the manuscript, rephrase the sentence "In addition, there as the absence of a prior sample size calculation [...]". While it is clear what is meant, the sentence does not fully make sense. You could do a retrospective effect size calculation, stating which effect sizes the study would be powered to detect with the achieved sample size. (Note: The key is not to compare that to the estimated ORs; e.g. if the minimum powered effects size is 1.9 and you report an OR of 2.1, that does not necessarily mean that that finding was powered or another one where the point estimate is 1.5 is not necessarily underpowered.) Or you could simply state that the study was not formally powered for the analyses that were done. But doing or not doing a sample size calculation in itself does not impact on study power -- what does is the actual recruitment.

TYPOS & SYNTAX

 This is a bit tricky as there is no line numbering with Wellcome Open Research, which would make it much easier to refer to the lines where the typos occurred. Hopefully the indications below are clear.

 * About half-way down in the third paragraph of the introduction section, remove "Though," from the start of the sentence.

 * About half-way down in the third paragraph of the introduction section, remove "On the contrary," from the start of the sentence.

 * In the second to last sentence of the third paragraph of the introduction section: "but there were no statistically increased incidence" -- the 'were' should be 'was'.

 * 'Containment and control measure' section title: that should probably be a plural ("measures")?

 * 'Data analysis' section, "Rubin's rule" should be plural: "Rubin's rules".

 * Third paragraph in the 'Results' section: 'three doses in TB groups' --> 'three doses in the TB groups' (missing preposition 'the')

 * Fifth paragraph of section 'Description of COVID-19 infection', "severer" --> "severe"

 * Table 3; I would clarify the levels for the vaccination status variables: "None", "1 dose", "2+ doses" rather than "None", "1" and "2".

 * 2nd paragraph of the 'Discussion' section: "[...] more common in non-TB [...]" --> "[...] more common in the non-TB [...]" (missing preposition 'the')

 * 2nd paragraph of the 'Discussion' section: "In TB patient's cohort [...]" --> "In the TB patients cohort [...]" (missing preposition 'the' and patients not patient's)

 * 3rd paragraph of the 'Discussion' section: "statistically significant increase in the mortality or disease severity" --> "statistically significant increases in mortality or disease severity" (plural for increase and a superfluous 'the' preposition)

 * 3rd paragraph of the 'Discussion' section: "particularly when active case finding [...] was taken place in TB patients" --> "particularly through active case finding [...] in TB patients"

 * 4th paragraph of the 'Discussion' section: "some other studies" --> "other studies"

 * 4th paragraph of the 'Discussion' section: "which could be probably due to the active screening of TB patients were diagnosed earlier" --> "which could be due to the active screening of TB patients who, as a result, were diagnosed earlier".

Is the work clearly and accurately presented and does it cite the current literature?

Yes

If applicable, is the statistical analysis and its interpretation appropriate?

Partly

Are all the source data underlying the results available to ensure full reproducibility?

Partly

Is the study design appropriate and is the work technically sound?

Yes

Are the conclusions drawn adequately supported by the results?

Yes

Are sufficient details of methods and analysis provided to allow replication by others?

Partly

Reviewer Expertise:

I am a biostatistician working mostly in infectious, tropical disease applications, including TB and SARS-CoV-2 / COVID-19 research.

I confirm that I have read this submission and believe that I have an appropriate level of expertise to confirm that it is of an acceptable scientific standard, however I have significant reservations, as outlined above.

# Response

**Published:** 2023-10-25

**Authors:** Aung Pyae Phyo

**Affiliations:** Shoklo Malaria Research Unit, Myanmar Oxford Clinical Research Unit, Myanmar

Thank you very much for your time and thorough suggestions. We genuinely appreciate your valuable feedback. We have carefully considered each of your comments and have made the necessary revisions to our paper accordingly. Your insights have been immensely helpful in improving the quality of our work.

 COMMENT R1: I would urge the authors to deposit their Stata analysis code on a code repository for easier reproducibility and removing any ambiguities about the specific analysis procedures that can be left from a text description of what was done.

 REPLY R1: Agreed. Please see the attached STATA do-file.

 COMMENT R2: I would similarly urge the authors to deposit the minimal dataset needed to reproduce the analyses from the present manuscript on a data repository. I understand that this may not be possible due to data policies and local regulations, in which case the authors could consider deriving a synthetic dataset based on the real analysis dataset. At the very least a direct link to request access for this particular dataset should be provided - the fewer hoops to jump through, the better.

 REPLY R2: We don’t provide the full patient dataset in “online open access” e.g., as supplementary files to a journal article nor through “External repository without case-by-case assessment” where MORU has no oversight or control of secondary uses made of the data. However, the full dataset can be requested from MORU data sharing committee **datasharing@tropmedres.ac** for the purpose of secondary or meta-analysis.

**STATISTICAL ANALYSIS & INTERPRETATION** Mostly the methods employed are clearly described, straightforward and appropriate. I do, however, have a few specific comments on the statistical analysis.

 COMMENT S1: Please provide full details of the ordinal logistic regression model that was used. I also did not see any discussion of model fit and diagnostics, but this is important given that ordinal logistic regression makes an assumption of proportional odds which should be checked.

 REPLY S1: Proportional odds assumption was checked with omodel command in Stata and the p-value was 0.68. Goodness of fit was assessed by ordinal version of the Hosmer–Lemeshow test using ologitgof command and the p-value was 0.93.

 COMMENT S2: While participants are recruited from 2 different TB clinics, and Table 1 shows that these 2 clinics differ at least in the TB patient: non-tb participant ratio, the analyses do not seem to account for clinic. Presumably there may be all kinds of other differences, not captured through measured variables, that may impact on outcomes, so I would suggest that the authors adjust for clinic in the analysis model or at least do a sensitivity analysis to investigate the effect of clinic on the study conclusions.

 Reply S2: We used cluster-robust standard error accounting for the clustering within the two clinics. We have updated the figures accordingly. See table 3.

 COMMENT S3: The authors make use of multiple imputation for the main analysis. This is great, but they should state clearly what imputation model was used with the MICE approach (presumably the Stata default, which would be PMM - but should be stated explicitly).

 REPLY S3: It is revised as following: “Multiple imputation using chained equations was conducted for Ct value and vaccination status using mi impute command for 20 times and combined by Rubin’s rules 24 using mi estimate command. All confounders, the outcome and study site were included in the imputation model. We used logistic regression for vaccination status, ordered (ordinal) logistic regression for severity grade, and truncated regression for Ct values (limiting to 0 to 45) for imputation. The observations with missing outcome (n=6) were used for imputation but were excluded from the analysis models.”

 COMMENT S4: The authors use a few times language that rules out an association or effect or difference (e.g. "TB was not associated with severe outcome" for the main study conclusion, "no difference between the two groups" for duration from vaccination to test positivity, "18.5 [...] which was not different from 18.8 [...]", ...). I think this language should be rephrased to make it clear that in this particular study there was not enough evidence to support such associations etc or that differences (e.g. 18.5 is not the same as 18.8; similar but not the same, so different) were not statistically significant. You do not have enough evidence to reject the null hypothesis in these cases, but that does not necessarily mean that the null is true.

 REPLY S4: Thanks for the suggestion. We have revised accordingly.

**MINOR COMMENTS**

 COMMENT M1:  The study involved 161 participants. But some variables seem to have missing data (e.g. 133 is the number of participants included in the complete case analysis, 155 is the denominator for COVID-19 vaccination status, 155 (though this is given as 156 in the abstract) for disease severity, ...), it would be helpful if Tables 1 and 2 could also list the amount of missing data for each variable.

 REPLY M1: Thanks for the suggestion. We have added accordingly.

 COMMENT M2:  In the third paragraph of the introduction, the sentence "The main finding was the similar pooled odds ratios in the COVID-TB group as compared to the non-TB “control” group COVID-19 infected patients: 2.21 (95% CI: 1.80, 2.70) for death and 2.77 (95% CI: 1.33, 5.74) for severe COVID-19 disease." is not fully clear. At least specify what groups the OR quoted here compares - since the confidence intervals exclude 1, one could read this to mean that TB very much increases the risk of death or severe disease, but clearly that is not what the authors meant to say here.

 REPLY M2: OR was 2.21 & 2.77 for death & severe disease in COVID-TB group, similarly higher than COVID patients without TB. Sorry for the confusion. We have corrected.

 COMMENT M3: In the 'Containment and control measure' section, if I read it correctly then controls got tested daily for COVID-19 as they reported to the clinic but TB patients were only tested upon admittance? OR does 'active screening' mean continuous testing during the stay in the clinic. Maybe good to clarify how often TB and non-TB participants were tested for COVID-19.

 REPLY M3: TB patients were tested upon admission and whenever they got symptomatic or contact with COVID confirmed patient.

 COMMENT M4: In the 'Data analysis' section, specify when the Chi-squared and when the Fisher test was used and/or indicate in a footnote to Tables 1 and 2 which test was used when.

 REPLY M4: We use Fisher’s exact test for table 1. Corrected accordingly. COMMENT M5: Please give a reference for Rubin's rules in the 'Data analysis' section. REPLY M5: We have added the citation.

 COMMENT M6: For Tables 1 and 2, it would also be great to see results stratified by clinic -- maybe as supplementary material tables? REPLY

 M6: Please see above. There was no apparent difference in clinical management between two clinics.

 COMMENT M7: Table 1 and third paragraph of the results section: might be worth to also state the overall p-value for comparing co-morbidity between TB and non-TB participants (Table 1 only formally compares each specific co-morbidity while the text states any comorbidity but does not give a p-value). I am not too fussed about getting a p-value but given that p-values are calculated for most other things, it would be consistent to give one here too.

 REPLY M7: Added as per suggestion.

 COMMENT M8: Table 1, unclear why no comparison (p-value given as "NA") is made between TB and non-TB participants in distribution of first dose of COVID-19 vaccine.

 REPLY M8: P value is added.

 COMMENT M9: Last sentence of the third paragraph of the 'Results' section reads "Only 2 out of the 104 TB patients (1.9%) were diagnosed as TB after COVID-19". This is a bit puzzling as by design the TB patients would have been recruited based on their TB status (as patients of the clinics). Presumably either some patients had their TB identified as a result of showing up to a test centre for COVID-19 then got admitted or else some of the caregivers or medical staff became TB positive during the study. Might be worth clarifying what happened here.

 REPLY M9: We have re-phrased for clarity.

 COMMENT M10: Table 2 for case classification at COVDI-19 diagnosis: unclear why each level is compared between groups rather than comparing the entire distribution of diagnosis levels (as done for other categorical variables with mutually exclusive levels in the very same table). I would recommend just giving a single p-value for comparing all levels between the 2 groups.

 COMMENT M11: p-values are variable reported to 2 or 3 decimal digits. Please give them consistently to the same number of decimal digits.

 REPLY M10 & 11: Revised accordingly.

 COMMENT M12: I would probably put the case classification at COVID-19 diagnosis and the worst clinical severity during the disease course right next to each other in the table rather than having other variables summarised between them.

 REPLY M12: Revised accordingly.

 COMMENT M13: Can you double-check the ORs for the vaccination status levels? While the CIs are large and include 1, the ORs are consistently >1, which would mean that 1 or 2+ doses of the vaccine had on average more severe COVID-19 disease than no vaccination (even if not statistically significant). This direction of effect seems puzzling. It may be true, but worth double-checking.

 REPLY M13: We have checked the vaccination records again and defined the vaccination status as effective when a person received two or more doses 14 days or more prior to the infection. Updated results are presented in the revised manuscript.

 COMMENT M14: Second last paragraph of the manuscript, rephrase the sentence "In addition, there as the absence of a prior sample size calculation [...]". While it is clear what is meant, the sentence does not fully make sense. You could do a retrospective effect size calculation, stating which effect sizes the study would be powered to detect with the achieved sample size. (Note: The key is not to compare that to the estimated ORs; e.g. if the minimum powered effects size is 1.9 and you report an OR of 2.1, that does not necessarily mean that that finding was powered or another one where the point estimate is 1.5 is not necessarily underpowered.) Or you could simply state that the study was not formally powered for the analyses that were done. But doing or not doing a sample size calculation in itself does not impact on study power -- what does is the actual recruitment.

 REPLY M14: Thank you for your comment. Some might argues against the sample size calculation for secondary data analyses (as is discussed here https://www.ncbi.nlm.nih.gov/pmc/articles/PMC7286546/), and in our case, analyses have been completed, so we decide not to conduct sample size calculation retrospectively at this stage. We have deleted this sentence from the Limitations, particularly because generalizability is not affected by the lack of sample size calculation.

**TYPOS & SYNTAX**

 This is a bit tricky as there is no line numbering with Wellcome Open Research, which would make it much easier to refer to the lines where the typos occurred. Hopefully the indications below are clear.

 * About half-way down in the third paragraph of the introduction section, remove "Though," from the start of the sentence. **Done**

 * About half-way down in the third paragraph of the introduction section, remove "On the contrary," from the start of the sentence.

 * In the second to last sentence of the third paragraph of the introduction section: "but there were no statistically increased incidence" -- the 'were' should be 'was'. **Done**.

 * 'Containment and control measure' section title: that should probably be a plural ("measures")? **Done**

 * 'Data analysis' section, "Rubin's rule" should be plural: "Rubin's rules". **Done**

 * Third paragraph in the 'Results' section: 'three doses in TB groups' --> 'three doses in the TB groups' (missing preposition 'the') **Done**

 * Fifth paragraph of section 'Description of COVID-19 infection', "severer" --> "severe" **Done**

 * Table 3; I would clarify the levels for the vaccination status variables: "None", "1 dose", "2+ doses" rather than "None", "1" and "2". **Changed**.

 * 2nd paragraph of the 'Discussion' section: "[...] more common in non-TB [...]" --> "[...] more common in the non-TB [...]" (missing preposition 'the') **Done**.

 * 2nd paragraph of the 'Discussion' section: "In TB patient's cohort [...]" --> "In the TB patients cohort [...]" (missing preposition 'the' and patients not patient's) **Done**

 * 3rd paragraph of the 'Discussion' section: "statistically significant increase in the mortality or disease severity" --> "statistically significant increases in mortality or disease severity" (plural for increase and a superfluous 'the' preposition) **Done**

 * 3rd paragraph of the 'Discussion' section: "particularly when active case finding [...] was taken place in TB patients" --> "particularly through active case finding [...] in TB patients" **Done**

 * 4th paragraph of the 'Discussion' section: "some other studies" --> "other studies" **Removed**

 * 4th paragraph of the 'Discussion' section: "which could be probably due to the active screening of TB patients were diagnosed earlier" --> "which could be due to the active screening of TB patients who, as a result, were diagnosed earlier". **Revised**
